# Integrated photonic emitter with a wide switching range of orbital angular momentum modes

**DOI:** 10.1038/srep22512

**Published:** 2016-03-03

**Authors:** Yu Wang, Peng Zhao, Xue Feng, Yuntao Xu, Kaiyu Cui, Fang Liu, Wei Zhang, Yidong Huang

**Affiliations:** 1 Department of Electronic Engineering, Tsinghua National Laboratory for Information Science and Technology, Tsinghua University, Beijing, China

## Abstract

Due to the nature of infinite dimensionality, the orbital angular momentum (OAM) has been considered as a new degree of freedom of light and widely expanded the scopes of substantial optical applications such as optical telecommunication, quantum information, particle manipulation and imaging. In recent years, the integrated photonic OAM emitters have been actively investigated due to both compactness and tunability. Essentially, the number of available OAM modes by dynamic switching should be large enough so that the dimensionality of OAM could be explored as much as possible. In this work, an integrated photonic emitter with a wide switching range of OAM modes is theoretically developed, numerically simulated, and experimentally verified. The independence of the micro-ring cavity and the scattering unit provides the flexibility to design the device and optimize the performance. Specifically, the dynamic switching of nine OAM modes (*l* = −4 ~ 4) with azimuthal polarization has been demonstrated by electrically controlled thermo-optic effect.

Since introduced by Allen in 1992^1^, the orbital angular momentum (OAM) of light has been recognized to be independent with the spin angular momentum (SAM) and constitute a complete orthogonal basis as a new degree of freedom^2^,^3^,^4^. The topological charge of the OAM mode, which is usually denoted as *l*, could be valued from any integer with infinite dimensionality. Such unique nature makes OAM an extremely attractive tool for optical telecommunication^5^,^6^,^7^ and quantum information^8^,^9^,^10^ since the system dimensionality can be significantly expanded. Specifically, more OAM modes involved indicates a higher data rate for optical telecommunication and a larger orthogonal basis for quantum information. OAM modes with different topological charges also have diverse characteristics of optical forces and phase variations, which are widely applied in particle manipulation^11^,^12^,^13^ and imaging^14^. In all the above applications, it is the dimensionality of available OAM modes that actually matters. Thus a wide switching range of OAM modes is extraordinary crucial for real applications.

More recently, as a combination of silicon photonics and OAM, integrated emitters have been actively investigated, showing inspiring superiorities of being compact and enabling dynamic switching^15^,^16^,^17^,^18^,^19^,^20^,^21^,^22^,^23^,^24^. Among the previous proposals, generating OAM modes has been mainly based on the micro-ring cavities^15^,^16^,^18^,^19^,^20^ by utilizing the phase gradient of the fundamental whispering gallery mode (WGM). And the dynamic switching between different OAM modes could be achieved through thermo-optic effect and plasma-dispersion effect^18^,^23^,^25^. It is worth to mention that the opened-ring structure in ref. 22 does generate both integral and fractional OAM modes. However, it is very hard to practically control a precise integral mode as the vast majority of the modes is fractional and the integral OAM modes are just a very tiny subset of the whole, especially when considering the possible fabrication errors. As for practical quantum and telecommunication applications, a pure integral OAM mode is quite essential, while fractional OAM mode would suffer from detection, propagation, orthogonality and so on. With micro-ring cavities, the pure integral OAM modes can be naturally selected due to resonance.

In this work, an integrated photonic emitter with a wide switching range of OAM modes is theoretically developed, numerically simulated, and experimentally verified. In our design, the micro-ring cavity and the scattering unit are spatially separated so that they could be optimized independently. A larger micro-ring cavity is beneficial to achieve a wider switching range, so the radius of 200 μm is adopted to achieve the dynamic switching of nine OAM modes (*l* = −4 ~ 4) with electrically controlled thermo-optic effect. Meanwhile, the scattering unit named cobweb is designed to efficiently and vertically scatter lightwaves around 1550 nm. Although only azimuthally polarized vectorial OAM mode is demonstrated in current design, it is also discussed that how the scattering unit could be further modified to achieve polarization diversity and unidirectionality of emission.

## Results

### Principle

The silicon micro-ring cavity is a basic functional unit since various photonic integrated devices can be implemented based on it^26^. The fundamental mode of a micro-ring cavity is so-called WGM, which has an annular and uniform intensity distribution around the center. For the WGM, the phase shift is azimuthally varied (ascending or descending depends on the propagating direction) with a constant phase gradient. Thus, with equidistantly placed gratings^16^,^18^, an OAM mode can be scattered from the ring cavity into free space with several periodic azimuthal phase evolutions from 0 to 2*π*. The number and direction of the periodic evolutions, thus the topological charge, are resulted from the phase shift between any two adjacent gratings, which could be determined by the WGM order in micro-ring cavity. Consequently, varying the WGM order in the micro-ring cavity could achieve the switching between different OAM modes^16^,^18^,^23^.

**Figure 1:**
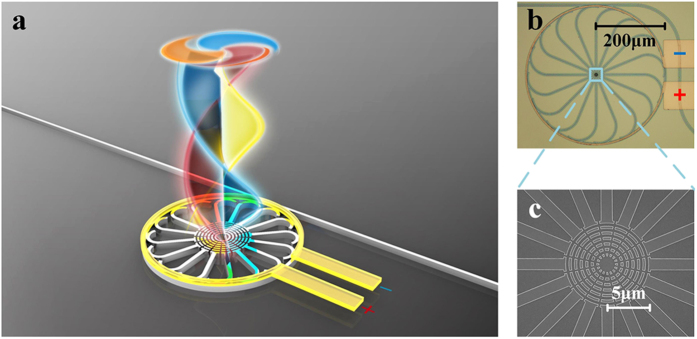
The integrated photonic emitter. (**a**) The schematic structure, (**b**) the micrograph and (**c**) the scanning electron micrograph of the fabricated device from top view.

Following the above discussion as well as our previous work^18^,^19^, a tunable OAM emitter is proposed and demonstrated in this work. As schematically shown in Fig. 1(a), there are a bus waveguide and a micro-ring cavity with 16 downloading waveguides combined with 16 gratings at the end. The whole scattering unit of all 16 gratings just looks like a cobweb. Figure 1(b,c) illustrates the micrograph and the scanning electron micrograph of the fabricated device from top view. An annular heating electrode is fabricated above the micro-ring cavity to vary the WGM order with thermo-optic effect^25^. The incident wavelength is considered to be around 1550 nm, as a typical choice for optical information carrying.

Partial power of the WGM is coupled to the downloading waveguides towards the gratings at the micro-ring center. As fully discussed in ref. 18, the phase shift between any two adjacent gratings would determine the topological charge and could be expressed as:


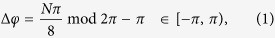


where *N* stands for the WGM order within the micro-ring cavity. Hence, there should be 16 possible values for 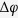 as 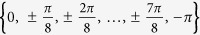. Basically, the topological charge of an OAM mode is simply defined as integrating the phase gradient along a closed path around the center (singularity) then dividing by 2*π*, which signifies that the topological charge stands for the number of phase periodic evolutions from 0 to 2*π* along the azimuthal direction^27^. Intuitively, there are 16 possible values for the topological charge. However, when 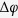 equals to 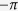, the scattered lights would be an azimuthally standing wave, which is the so-called cogwheel beam and possesses total OAM of zero^13^,^28^. As a result, by excluding this particular situation, the topological charge could only have 15 values as:


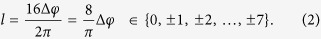


Compared with some other previous work^15^,^16^,^20^, in which the scattering unit for lightwaves is right on or adhered to the micro-ring cavity, the most significance of our design is to separate the micro-ring cavity from the scattering unit since such two components, actually, play relatively different but crucial roles for generating the desired OAM modes. The micro-ring cavity provides the phase shift between the gratings and results different topological charge with varied WGM order. The scattering unit at the micro-ring center, meanwhile, would determine the beam characteristics, such as polarization, focusing, beam waist, etc. Therefore, these two functionalities could be handled and optimized independently. In the next section, the designing of both the ring cavity and scattering unit would be discussed in detail. For the micro-ring cavity, the radius is as large as 200 μm to achieve the dynamic switching of nine OAM modes (*l* = −4 ~ 4). On the other hand, the scattering unit is designed to effectively and vertically scatter lightwaves around 1550 nm with azimuthal polarization.

### Design and simulation

Thanks to the independence of the micro-ring cavity and the scattering unit, we could first focus on the design of the micro-ring cavity. As shown in equation (1, 2), the topological charge of OAM modes could be tuned by varying the WGM order in micro-ring cavity, which is given by


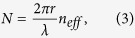


where *r* stands for the radius of micro-ring cavity, *λ* stands for the operating wavelength (in vacuum), and *n*_*eff*_ stands for the effective refractive index of silicon (valued as 2.6 approximately for both silicon stripe and ridge waveguides on silicon-on-insulator substrate). Both *r* and *λ* are relatively fixed in a fabricated OAM emitter, the preferred solution to implement the dynamic switching is by varying *n*_*eff*_ through thermo-optic effect or plasma-dispersion effect^25^. Therefore, the switching range of topological charge (rounded down) is given by


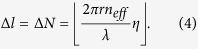


**Figure 2:**
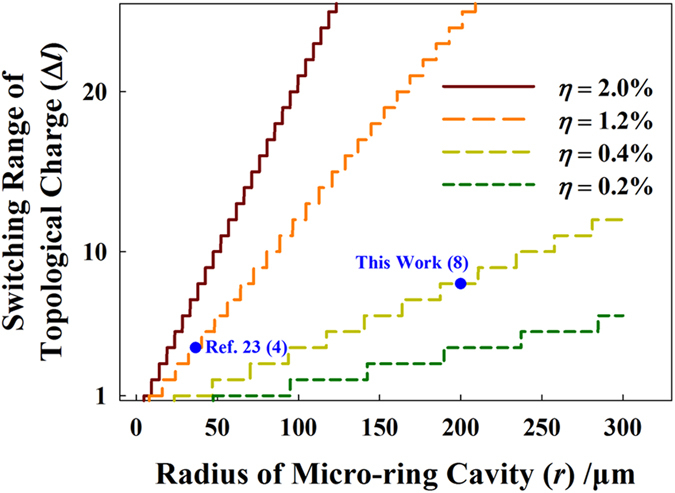
The switching range of topological charge versus the radius of micro-ring cavity with different modulation ratio.

Here, a parameter of the modulation ratio 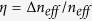 is introduced to evaluate the variation extent of *n*_*eff*_. Generally, a larger modulation ratio is related to a higher temperature in thermo-optic effect or a higher voltage on the PN junction in plasma-dispersion effect. The common maximum modulation ratios in thermo-optic and plasma-dispersion effects are of the orders of 1% and 0.1%, respectively^25^,^29^,^30^. Figure 2 illustrates the relation between the switching range of topological charge and the radius of micro-ring cavity, with different modulation ratio. As shown in Fig.2, the switching range is linearly proportional to the radius and the slope is related to the modulation ratio. Obviously, a larger micro-ring cavity is helpful to achieve a wider switching range. Intuitively, a larger micro-ring cavity implies a smaller free spectrum region so that a larger variation span of resonant peaks, as well as their corresponding WGM orders, could be covered under the same modulation ratio. The results of this work 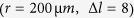 and ref. 23 (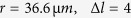,) are also marked in Fig. 2. It could be found that the wider switching range is achieved mainly due to the larger micro-ring cavity.

After the micro-ring cavity is settled, the scattering unit would be designed. Both the Finite Difference Time Domain (FDTD) method and its integral form of Finite Integration Technique (FIT) method have been employed in our simulation. The quasi-TE mode is assumed to be excited at only one downloading waveguide outside the scattering unit. Then each output of the 16 gratings could be obtained by adding a corresponding phase shift according to the grating position. Finally, the output of the whole scattering unit is considered as the linear combination of all 16 gratings due to the linear nature of electromagnetic waves.

**Figure 3:**
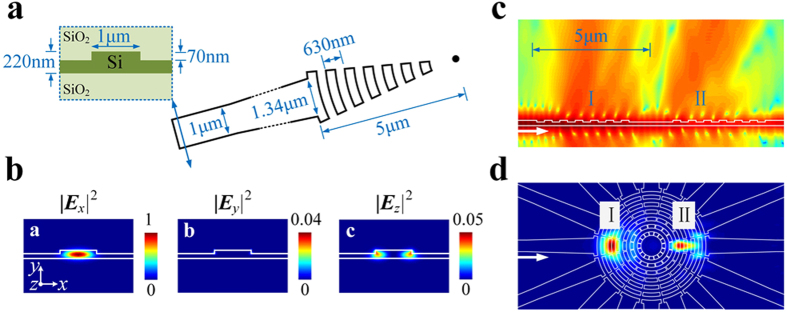
The characteristics of one grating. (**a**) The structural parameters (not for scale), (**b**) the electric field of the fundamental quasi-TE mode propagating along the ridge waveguide, (**c**) the lateral and (**d**) the top view of the intensity distribution of scattered lightwave.

Figure 3(a) illustrates the structural parameters of one grating at the micro-ring center. To reduce the transmission loss, the ridge waveguide is employed in our design. The period of gratings is designed as 630 nm with duty cycle of 50%, which is quite suitable and efficient for scattering the lightwave around 1550 nm vertically. Figure 3(b) illustrates the calculated electric field of the fundamental quasi-TE mode propagating along the ridge waveguide, in which the transversal *x* component dominates. Thus, the scattered OAM mode would be azimuthally polarized vectorial beam. Figure 3(c,d) illustrate the lateral and the top view (~2 μm up from the grating surface) of the intensity distribution of scattered lightwave from one grating. Though most power is firstly scattered by the grating at the end of download waveguide (position I), the residual power would keep on propagating and be secondly scattered by the grating located at the dual side (position II). As the phase relation of all the secondly scattered lightwaves is the same as that of the firstly scattered ones, both of them would possess the same topological charge.

**Figure 4:**
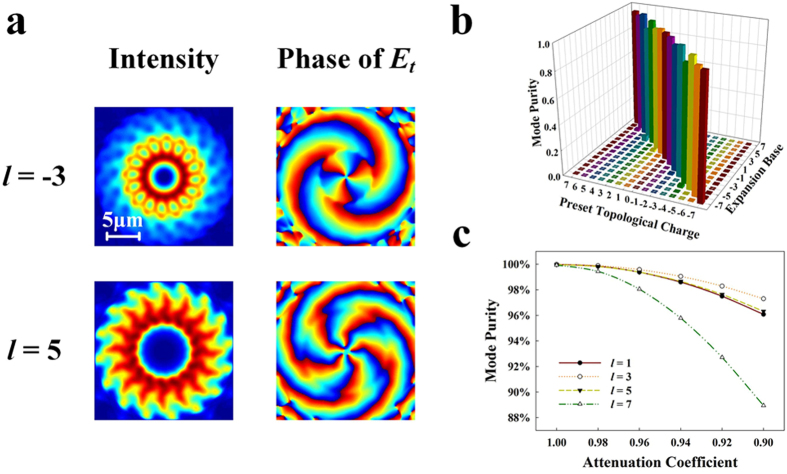
The characteristics of OAM modes. (**a**) The intensity and phase profiles with *l* = −3 and *l* = 5. (**b**) The calculated mode purity of OAM modes with *l* = −7 ~ 7 with equation (6). (c) The mode purity versus attenuation coefficient.

By azimuthally duplicating the obtained electromagnetic field with proper phase shifts, OAM modes with determined topological charge could be generated. Figure 4(a) illustrates intensity and phase (of the azimuthal component ***E***_*t*_) profiles of two examples with settled topological charge of −3 and 5. Due to the different settled phase shifts, pure OAM modes with different topological charges are generated and could be easily verified from the phase profiles. Unsurprisingly, OAM modes of other topological charges have similar characteristics and are therefore not listed here for simplicity.

For a real device, however, the attenuation in the micro-ring cavity has to be taken into consideration. As the WGM propagates along the azimuthal direction, the power would be attenuated because of propagating loss and coupling loss to download waveguide. Consequently, the power of scattered light from each grating is not unique but exactly as a geometric sequence. Due to the low propagating loss of ridge waveguide (~2 dB/cm), only the coupling loss needs to be considered in this case. Here the attenuation coefficient of 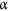 is used to represent percentage of the residual power after a downloading waveguide and the value is 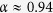 in our design (the calculation detail is shown in the Method section). The attenuation would distort both the intensity and phase profiles of generated OAM mode. To numerically evaluate the distortion, the mode purity analysis is employed^20^. The azimuthally polarized OAM mode could be decomposed by Fourier expansion as


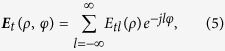


where 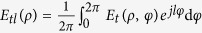. Then the mode purity of topological charge *l* is defined as


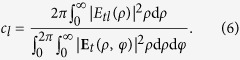


Figure 4(b) illustrates the mode purity of OAM modes with *l* = −7 ~ 7, where the mode is Fourier-expanded respect to a finite basis also from −7 to 7. Mode purity values that belong to the same preset topological charge are normalized by their sum. Results show that the mode purity of the desired topological charge would be mostly above 95% with a maximum of 99% for *l* = ±3 and a minimum of 91% for *l* = ±4, indicating that the OAM modes are of both high quality and purity. Furthermore, when considering the possible fabrication errors in a real situation, the mode purity of the desired topological charge under different attenuation coefficient 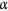 is also investigated and shown in Fig. 4(c). The mode purity declines with decreasing attenuation coefficient and OAM modes with higher topological charges are more sensitive to the attenuation. But, even when the attenuation coefficient drops to 0.90, the purity of most OAM modes exceeds 90%. Consequently, our proposal shows good robustness to the possible fabrication errors.

### Measurement

The proposed device is fabricated following the procedure in the Method section. As aforementioned, the generated OAM mode is azimuthally polarized and could be decomposed into two spin parts as follows^16^,^22^


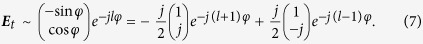


Here, 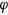 stands for the azimuthal coordinate in the cylindrical coordinate system. The carried SAM and OAM per photon of left-handed circular polarized (LHCP) part would be −*ћ* and (*l* + 1)*ћ*, while those of the right-handed circular polarized (RHCP) part would be *ћ* and (*l*−1)*ћ*. The value of *l* is then observed by interfering the generated beam with a coherent LHCP (RHCP) beam as reference. The interference pattern would be a spiral figure of *l* + 1 (LHCP) or *l* − 1 (RHCP) arms.

**Figure 5:**
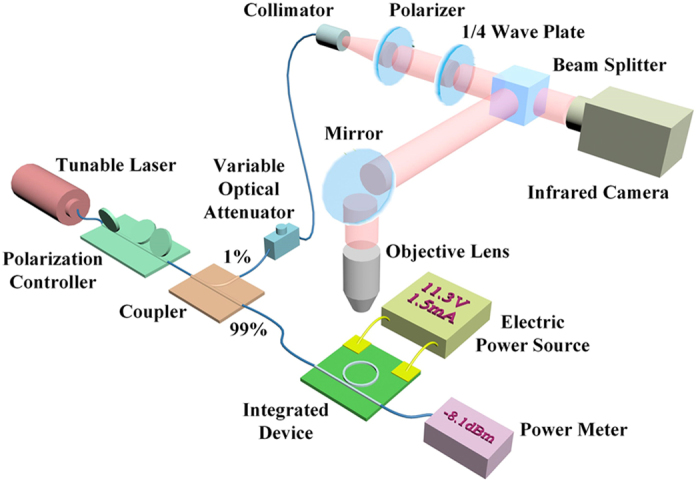
The schematic diagram of the measuring system.

Figure 5 illustrates the schematic diagram of the measuring system. The polarization controller is to ensure a quasi-TE mode in waveguide. Due to the large coupling loss between the lensed fiber and the waveguide, 99% power of the incident beam is connected to the lensed fiber and then coupled to the integrated device, while the rest is tuned to a LHCP or RHCP beam as reference. An additional variable optical attenuator is further adopted to balance the powers of the two paths. The electric power source is applied on driving the electrode and heating the silicon micro-ring cavity in success.

**Figure 6:**
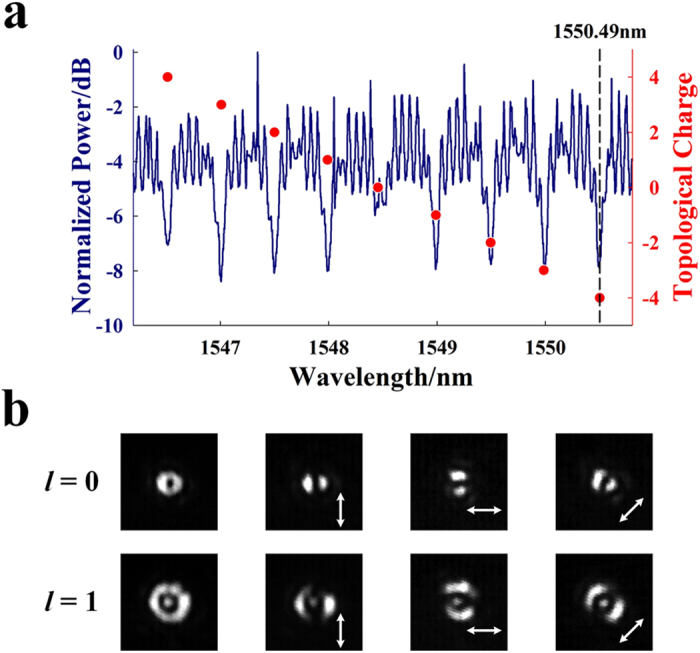
Static characteristics. (**a**) The normalized transmission spectrum and (**b**) the polarization state of the OAM modes, indicating that they are azimuthally polarized vectorial beams.

The static characteristics of the device are firstly investigated with various incident wavelengths. The normalized transmission spectrum of the emitter is shown in Fig. 6(a). The small but obvious fluctuations on the measured spectrum are caused by the mirror-like reflection between the input and output ends of the waveguide. As each WGM corresponds to a unique topological charge, the values of topological charges are also marked, regarding to the resonant wavelengths. Furthermore, the polarization state of generated OAM mode is examined by rotating the direction of a polarizer before the infrared camera. As shown in Fig. 6(b), the intensity distribution at wavelength of 1548.49 nm (*l* = 0) and 1549.99 nm (*l* = −1) could be greatly varied following the orientation of polarizer, which indicates that azimuthally polarized vectorial beams are successfully generated as expected. The experimentally measured emitting angle after the objective lens is around 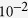 degrees and the measured power after the objective lens is around 1~5% of that injected into the bus waveguide. The efficiency of our microscope system is estimated as ~40% so that the emission efficiency of the whole device is about 2.5~7.5%. In our grating design, emission efficiency is almost the same for the upper and lower half-space. Thus, the ideal value of maximum efficiency is ~50% since only the emission of upper half-space is collected. Compared with ideal maximum efficiency, the emission efficiency can be further improved by optimizing designs and fabrications.

**Figure 7:**
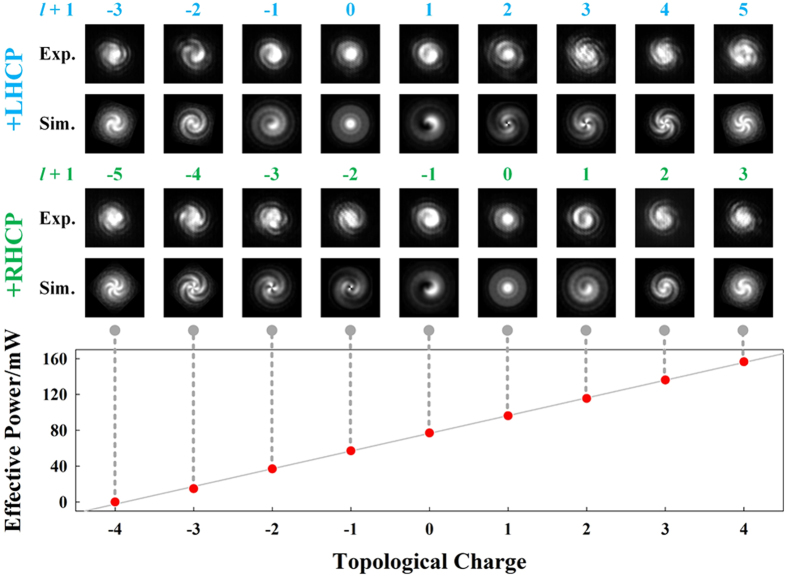
Dynamic characteristics with incident wavelength fixed at 1550.49 nm. Dynamic switching of nine OAM modes has been implemented with the effective switching power of 20 mW per mode.

Next, the dynamic characteristics of the device are investigated with the incident wavelength fixed at 1550.49 nm. By continuously increasing the driving voltage applied on the electrode, the local temperature of micro-ring cavity would be increased. Thanks to the large thermo-optic coefficient of silicon material^25^, the effective refractive index *n*_*eff*_ would be significantly varied and the transmission spectrum would be red-shifted. Meanwhile, the WGM order and the resulting topological charge of OAM mode would increase. Both the experimental and simulated (without considering the attenuations) results are shown in Fig. 7. The number and spiral direction of the interference fringes indicate the value and sign of topological charge. Then under meticulously controlled driving voltage, different OAM modes could be generated at a certain resonant peak. With a maximum modulation ratio of only 0.4% (*l* = 4, the last column in Fig. 7), nine OAM modes can be dynamically tunable. Eliminating the resistance of contacting electrode (see the Method for more details), the power on the heating electrode is referred as the effective power. We then conclude the linear dependence between the topological charge of OAM mode and the effective power in Fig. 7. Result of linear fitting shows that the effective switching power of the emitter is about 20 mW per mode.

## Discussion

As discussed and demonstrated above, a larger micro-ring cavity should be adopted to achieve a wider switching range of OAM modes. Intuitively, the power consumption maybe increases with a larger device. To investigate how the size of micro-ring cavity affects the power consumption, we assume that only the effective power is considered and the concept of effective power density per unit length of heating electrode (*P*_0_) is introduced here. As one can see from Fig. 7, the switching range of topological charge is linearly dependent on the effective power 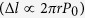. Meanwhile, the switching range of topological charge is also linearly proportional to the modulation ratio 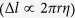, as shown by equation (4). It implies that, in a certain but large enough temperature range, the relation between the modulation ratio and the effective power density is also linear 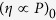. Enlarging the radius of micro-ring cavity and the annular heating electrode 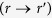 would reduce the effective power density 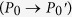 and modulation ratio 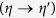 at the same time. Under the same heating electrode in terms of width and height, the effective power density and the resulting modulation ratio inversely would scale with the increasing radius 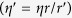. Amazingly, reducing modulation ratio could be completely compensated by increasing radius as their product does remain unchanged 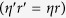. Following equation (4), the switching range of topological charge keeps constant 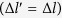. Thus we could draw the conclusion that a larger micro-ring cavity does not necessarily bring about a lager switching power consumption per mode. Instead, the switching power consumption remains unchanged.

An annular electrode is fabricated on the micro-ring cavity in this work. According to a previous work^31^ and ref. 23, the switching speed would be 10~15 KHz. If a higher switching speed is desired, plasma-dispersion effect of silicon should be adopted. For example, in ref. 32, modulation speed up to 10 GHz has been demonstrated with silicon 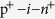 structure. And it is worth to mention that the low 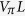 figure of merit of 0.36 V-mm could further reduce the size of micro-ring cavity. With the maximum applied voltage of around 40 V, which is the exactly experimental parameter in this work, the dynamic switching of nine OAM modes could be achieved in the micro-ring cavity with the radius of only 22.9 μm. The modulation speed of 42.7 GHz has also been demonstrated^33^. Furthermore, if one considers the polymer-silicon hybrid system for the micro-ring structure, the modulation speed has the potential to perform in the regime of 100 GHz or even higher^34^.

Due to the independence of the micro-ring cavity and the scatting unit in our proposed device, it is worth to mention that generating OAM modes with arbitrary polarization states would be close at hand with particularly designed grating structures. 2-D grating structures could be employed to scatter lightwaves with diverse polarizations^35^,^36^,^37^. By controlling the amplitudes and phase shift of two scatter lightwaves with perpendicular polarizations, OAM modes of arbitrary polarization states, such as azimuthal, radial, circular, as well as elliptical polarizations, could be generated in principle. In addition, if blazed structures are employed for grating design, the unidirectional emission of OAM modes could be achieved^38^,^39^ to enhance the emission efficiency.

As a matter of fact, limited number of gratings would reduce the angular resolution and available number of the OAM modes, as the more the gratings, the better the situation. However, our design does not necessarily mean that we could only have 16 gratings around the center. By adopting a smaller sized grating and a larger radius of the whole scattering unit, much more gratings could be achieved, *e*.*g.*, 64 or even more. Besides varying the coupling length, another method is varying the gap distance between the bus waveguide and micro-ring cavity to well balance the power in each downloading waveguide. Obviously, this issue would be more significant if more gratings are utilized to achieve a high-order OAM emission and a more precise fabrication process is therefore required. We are still working on it now.

In summary, the integrated photonic emitter is proposed and demonstrated in this work. As the micro-ring cavity and the scattering unit could be optimized independently, a large micro-ring cavity for a wide switching rang and a scattering unit for azimuthal polarization are adopted. By further utilizing the above special characteristics of our design, we believe that OAM modes with polarization diversity^40^,^41^,^42^, unidirectionality of emission and a much wider switching range could be achieved to bring far more potentials in applications of optical telecommunications^17^,^18^,^24^, quantum information^43^, particle manipulation^44^, and imaging^45^.

## Method

### Structural parameters and attenuation coefficient

The emitter is designed on a silicon-on-insulator substrate in which the thickness of the top silicon layer is 220 nm and that of the buried oxide layer is 3 μm. The ridge waveguide is adopted to achieve low transmission loss. The width and height of the ridge are 1 μm and 70 nm, respectively. The radius of micro-ring cavity is 200 μm and the radius of downloading waveguide is 50 μm. The gap between the bus waveguide and the micro-ring cavity is around 230 nm, while the gap between the micro-ring cavity and the downloading waveguide is 350 nm.

**Figure 8:**
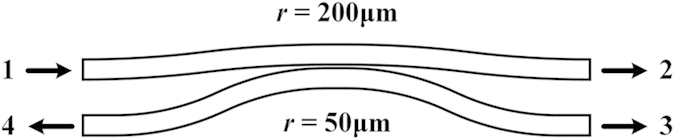
The coupling section in the emitter.

The relatively large gap between the micro-ring cavity and the downloading waveguide ensures a very weak coupling so that the scattered power at each grating could be balanced as much as possible. In order to numerically determines the value of 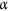, the coupling section is particularly studied as shown in Fig. 8. The incident wavelength at port 1 is set to be 1550 nm. The S-parameters could then be calculated and obtained as {S_11_ = 0.0002, S_21_ = 0.9371, S_31_ = 0.0405, S_41_ = 0.0000}. Therefore, the attenuation coefficient *α* is obtained as 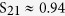.

### Fabrication

ZEP520A servers as the pattern mask in electron beam lithography (EBL) process, after which a mixture of O_2_ and 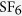 is employed to etch the silicon with inductive coupled plasma (ICP). In the following, another 600 nm-thick protection layer of 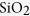 is developed on the top layer by plasma enhanced chemical vapor deposition (PECVD). The heating electrode of titanium and contacting electrode of aluminum are consecutively developed on the 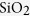 top by vacuum evaporation, with the thicknesses of about 100 nm and 300 nm respectively. The annular heating electrode above the micro-ring cavity is about 4 μm wide.

### Estimation of effective power

In order to investigate the resistances of both contacting electrode and heating electrode, several samples with different heating electrode parameters are fabricated and measured. With the same height of 100 nm for all the heating electrodes, the other two parameters (length, width) are given by: sample 1 (500 μm, 8 μm), sample 2 (3100 μm, 6 μm), sample 3 (1200 μm, 4 μm), and sample 4 (1200 μm, 3 μm). The corresponding resistances are 3000 Ω, 11000 Ω, 7600 Ω, and 9600 Ω, respectively. Thus the resistance of contacting electrode is calculated as ~1900 Ω. The total switching power per mode is about 26 mW. Then by subtracting the power on the contacting electrode, the effective power could then be obtained as ~20 mW.

## Additional Information

**How to cite this article**: Wang, Y. *et al*. Integrated photonic emitter with a wide switching range of orbital angular momentum modes. *Sci. Rep*. **6**, 22512; doi: 10.1038/srep22512 (2016).
